# RCC1 functions as a tumor facilitator in clear cell renal cell carcinoma by dysregulating cell cycle, apoptosis, and EZH2 stability

**DOI:** 10.1002/cam4.6594

**Published:** 2023-09-25

**Authors:** Yunfei Wu, Zhijie Xu, Xiaoyi Chen, Guanghou Fu, Junjie Tian, Baiye Jin

**Affiliations:** ^1^ Department of Urology, The First Affiliated Hospital, School of Medicine Zhejiang University Hangzhou China; ^2^ Zhejiang Engineering Research Center for Urinary Bladder Carcinoma Innovation Diagnosis and Treatment Hangzhou China

**Keywords:** apoptosis, cell cycle, clear cell renal cell carcinoma, EZH2, RCC1

## Abstract

**Background:**

RCC1 functions as a pivotal guanine nucleotide exchange factor and was reported to be involved in mitosis, the assembly of the nuclear envelope, nucleocytoplasmic transport in cell physiological processes. Recent studies reported that RCC1 could regulate immunological pathways and promote the growth of some malignant solid tumors. However, the prognostic value and exact function of RCC1 remain unknown in patients with clear cell renal cell carcinoma (cRCC).

**Methods:**

The UALCAN and KM plotter portals were used to analyze the expression profile and related tumor prognosis of RCC1 in ccRCC using data from TCGA. The expression profile of RCC1 was also confirmed in clinical samples using qRT‐PCR, western blotting, and immunohistochemistry. The role of RCC1 on ccRCC cells in vitro was confirmed by a series of functional assays. Animal experiments were performed to verify the suppressive effect of RCC1 knockdown on tumor growth in vivo. The correlation of RCC1 expression with that of EZH2 was explored in clinical samples using IHC. The interaction between RCC1 and EZH2 was further verified using a CO‐IP assay and a protein stability assay.

**Results:**

RCC1 was upregulated in ccRCC tissues compared with normal tissues in TCGA dataset and paired clinical samples. RCC1 promoted ccRCC progression by accelerating the cell cycle and suppressing apoptosis. In addition, RCC1 could bind EZH2 and regulate its expression at the posttranscriptional level. RCC1 and EZH2 expression showed a strong correlation in clinical samples. Further investigation proved that RCC1 regulated EZH2 protein stability through the ubiquitin–proteasome pathway.

**Conclusions:**

RCC1 could be a potential therapeutic target in ccRCC. The RCC1/EZH2 axis takes part in the development of ccRCC.

## 1 INTRODUCTION

According to the data from CANCER STATISTICS 2023,^1^ around 81,800 people are newly diagnosed with renal cancer and around 14,890 people die of it annually. Clear cell renal cell carcinoma (ccRCC) is the most common pathological subtype of renal cell carcinoma (RCC) and accounts for 70%–80% of RCC cases.^2^ Due to the asymptomatic and insidious features of ccRCC, many patients are luckily detected at an early stage of tumor development as a result of annual routine physical and imaging examinations.^3^, ^4^, ^5^ Unfortunately, some patients seek medical advice only if they have symptoms such as hematuresis and abdominal pain and, therefore, are diagnosed when the tumor has already progressed to an advanced stage and may have metastasized.^3^, ^6^, ^7^ Surgery is still the primary treatment for ccRCC because of its intrinsic radiotherapy and chemotherapy resistance.^7^, ^8^ Recently, new targeted therapies (such as with tyrosine kinase inhibitors) and immunotherapies (such as with anti‐PD‐1/L1 and ‐CTLA4 antibodies) have shown efficacy in the treatment of metastatic ccRCC patients with unresectable ccRCC.^6^, ^9^, ^10^, ^11^ Therefore, further investigation of the mechanisms of ccRCC progression may reveal new drug targets and provide new treatment options and survival benefits for unresectable ccRCC patients.

Regulator of chromatin condensation 1 (RCC1) is a key guanine nucleotide exchange factor that promotes the accumulation of Ran GTPase.^12^, ^13^ It is universally acknowledged that RCC1 is involved in mitosis, the assembly of the nuclear envelope, nucleocytoplasmic transport in normal cell physiological processes.^14^, ^15^, ^16^ Recent studies reported that RCC1 regulates immunological pathways and promotes the growth of some malignant solid tumors.^17^, ^18^, ^19^, ^20^, ^21^ For example, in lung cancer, RCC1 was shown to regulate the tumor sensitivity to immunotherapy by controlling PD‐L1 expression.^17^ In glioblastoma, RCC1 knockdown promoted the effect of radiation on tumor stem cells.^18^ In addition, RCC1 expression had prognosis prediction capacity for colorectal liver oligo‐metastases.^20^ However, the prognostic value and precise function of RCC1 in ccRCC remain unknown.

Enhancer of zeste homolog 2 (EZH2), an indispensable subunit of polycomb repressive complex 2 (PRC2), binds to its target gene promoters and catalyzes the formation of H3K37me3 to repress gene expression.^22^, ^23^, ^24^ Numerous studies demonstrated that the overexpression of EZH2 is involved in the progression and metastasis of various malignant tumors, including ccRCC.^25^, ^26^, ^27^, ^28^ Some studies reported that in ccRCC, EZH2 regulates kinase reprogramming, leading to sunitinib resistance, and is a cancer stem cell marker.^27^, ^29^ In addition, the regulatory mechanisms of EZH2 expression were also extensively explored. At the transcriptional level, miRNA binding to the 3′UTR of EZH2 promoted its mRNA degradation.^30^, ^31^, ^32^ STAT3, a well‐known transcription factor, was shown to combine with the promoter of EZH2 and facilitate its transcription.^33^ At the posttranscription level, the stability of the EZH2 protein is regulated by multiple chemical modifications, such as phosphorylation, methylation, acetylation, O‐GlcNAcylation, and ubiquitylation.^22^ However, the role of EZH2 in ccRCC needs further investigation.

In this study, we verified the expression of RCC1 in datasets and clinical samples and found that RCC1 was overexpressed in ccRCC compared with adjacent normal tissues and its high expression was closely correlated with worse clinical outcomes. Moreover, RCC1 knockdown and overexpression influenced the proliferation capacity of ccRCC cells. Finally, we discovered that RCC1 regulated EZH2 expression through the ubiquitin–proteasome pathway, promoting ccRCC development and progression. Taken together, our results provide evidence for the possibility of developing therapies targeting RCC1 in ccRCC.

## 2 MATERIALS AND METHODS

### 2.1 Comprehensive analysis

The KM plotter^34^ was used to analyze and visualize the relationship between the mRNA expression of RCC1 and overall patient survival. The cutoff was defined using the option to “auto select best cutoff.”

UALCAN^35^ is a bioinformatics portal and was used to visualize the relationship between RCC1 expression (mRNA or protein) and various clinicopathological data in TCGA and CTPAC datasets.

The Cancer Genome Atlas (TCGA) database was used to analyze the correlation between RCC1 mRNA expression and different clinical features.

### 2.2 Cell culture and transfection

The ACHN, 786‐O, 769‐P, and A498 cell lines were bought from the cell bank of the Chinese Academy of Sciences. The 786‐O, 769‐P, A498, and ACHN cell lines were separately cultured in RPMI 1640 (786‐O, 769‐P) and MEM (minimum essential medium, A498, ACHN) in an incubator at 37°C, in the presence of 5% CO_2_. All media (Procell) contained 10% fetal bovine serum (Procell) and a 1% penicillin/streptomycin/amphotericin B mixed solution (Solarbio). Si‐NC and si‐RNA against RCC1 were purchased from Sunya Biological company. Si‐RNA was diluted to 20 μM before use. The sequences used were as follows: SiNC sense (UUCUCCGAACGUGUCACGUdTdT), si‐NC antisense (ACGUGACACGUUCGGAGAAdTdT); si‐RCC1#1 sense (UGGAGAUGAUGGGCAAACATT), si‐RCC1#1 antisense (UGUUUGCCCAUCAUCUCCATT); si‐RCC1#2 sense (GGCACAGAAUCUUGCUUCAUATT), si‐RCC1#2 antisense (UAUGAAGCAAGAUUCUGUGCCTT). The above si‐RNA sequences were mixed with the jetPRIME transfection reagent (Polyplus) before transfection into cells according to the manufacturer's protocol.

The sh‐RCC1 lentivirus and its corresponding negative control (sh‐NC), the OE‐RCC1 lentivirus with the Flag tag and its corresponding negative control (OE‐NC) and the EZH2‐pcDNA3.1‐3x‐FlAG‐C overexpression plasmid and its corresponding negative control (OE‐NC) were bought from Genechem company. The RCC1 sequence was cloned in the Ubi‐MCS‐3xFLAG‐CBh‐gcGFP‐IRES‐puromycin vector (GV492) to produce an RCC1‐overexpressing lentivirus. Stably transfected cell lines were constructed and selected by treating them with 5 μg/mL of puromycin (Sigma‐Aldrich; Merck KGaA) for 5 days.

### 2.3 Cell counting kit‐8 (CCK‐8), colony formation, and EDU assay

The A498 and 786‐O cells (pretreated with si‐NC or si‐RCC1) and the 769‐P cells (transfected with OE‐NC/OE‐RCC1) were seeded in 96‐well plates, adding 2000 cells per well. Next, on appointed Day 0, 1, 2, 3, and 4, the cells were mixed with 10% CCK‐8 reagent (MCE) and incubated for 1 hour in the presence of 5% CO_2_, at 37°C. Then, the value of OD 450 nm, indicating the relative cell viability, was measured using a Bio‐rad microplate reader.

For the colony formation assay, 1000 cells were plated into each well of 6‐well plates. After cultivation for 7–10 days, each well was washed using PBS (Procell) and stained with 4% paraformaldehyde and 0.5% crystal violet (Beyotime).

The EDU assay was also performed to detect cell viability using the Alexa Fluor 594 BeyoClick™ EdU Cell Proliferation Kit (Beyotime) according to the manufacturer's protocol.

### 2.4 Cell cycle and apoptosis

For the cell cycle assay, pretreated cells were mixed with 75% ethanol and placed at −20°C overnight. On the next day, the cell suspension was brought to room temperature, and the cells were subsequently washed with PBS and centrifuged at 200 *g* for 5 min. Then they were mixed with a cell cycle staining reagent (Multisciences) and incubated in the dark for 30 min. The cell cycle distribution of the cells was detected using BD FACSCanto™ II and analyzed using Modfit LT 5.0 (Verity Software House). For the apoptosis assay, the cells were harvested and incubated with 200 μL of binding buffer containing 2 μL of FITC and 4 μL of PI fluorescence reagent (Multisciences) for 10 min in the dark. The proportion of apoptotic cells was detected using BD FACSCanto™ II and analyzed using FlowJo 10.8.1 (Becton, Dickinson and Company).

### 2.5 Immunohistochemistry (IHC)

Adjacent normal tissues and renal cell carcinoma samples were collected from the archive of the Institute of Urology, the First Affiliated Hospital of Zhejiang University School of Medicine. All patients provided a written informed consent. Our study was carried out in accordance with the Declaration of Helsinki and approved by the Ethics Committee of the First Affiliated Hospital at Zhejiang University School of Medicine. The specific protocol for IHC was described in detail in a previous study.^36^ The IHC sections were incubated with an anti‐RCC1 antibody (Abclonal) overnight at 4°C and subsequently treated with a secondary antibody (Fdbio science) for 30 min at 37°C. Then, the sections were stained with DAB, counterstained with hematoxylin and subjected to alcohol dehydration. The IHC results were evaluated using a microscope. The staining score of each IHC section was calculated using a formula (score of the staining intensity multiplied by the score of the staining area). The staining area was evaluated as follows: a score of 0 indicated negative staining; a score of 1 indicated a staining of 1%–24%; a score of 2 indicated a staining of 25%–49%; a score of 3 indicated a staining of 50%–74% and a score of 4 indicated a staining of 75%–100%. As for the staining intensity, a score of 0 indicated negative staining, while positive staining was assigned a score according to its intensity, that is, a score of 1, indicated as “1+,” a score of 2, indicated as “2+,” and a score of 3, indicated as “3+.”

### 2.6 Western blotting (WB) and subcellular fractionation

The specific protocol of WB was described in detail in a previous study.^36^ ccRCC cells subjected to different treatments were lysed with RIPA lysis buffer containing 1% protease inhibitor cocktail (Fdbio). After concentration measurement, the protein samples were separated on 4%–20% Tris‐acetate gels (ACE Biotechnology) and then transferred onto PVDF membranes. The membranes were blocked in 5% non‐fat milk for 1 h before incubation with the primary antibodies at 4°C overnight. The next day, the membranes were incubated with the secondary antibodies for 1 h at room temperature after washing with TBST three times. The protein bands were detected by the EZ‐ECL chemiluminescence detection kit (Fdbio).The primary antibodies used in this study were anti‐RCC1 (1:1000, Abclonal), anti‐GAPDH (1:10000, Abcam), anti‐cleaved caspase‐3 (1:1000, CST, Danvers, MA, USA), anti‐P21 (1:1000, CST), anti‐P27 (1:1000, CST), anti‐CDK2 (1:1000, CST), anti‐EZH2 (1:1000, CST), and anti‐UB (1:1000, Abclonal).

According to the Nuclei and Cytoplasmic Protein Extraction kit (Beyotime) instruction, cytoplasmic and nuclear proteins were extracted to analyze the effect of RCC1 on EZH2 location. GAPDH was used as a cytoplasmic control protein, and Histone H3 (H3, Abcam) was used as a nuclear control protein.

### 2.7 Quantitative real‐time PCR (qRT‐PCR)

Total RNA was isolated from frozen adjacent normal tissues and renal cell carcinoma samples using the TRIzol protocol (Invitrogen; Thermo Fisher Scientific). Total RNA was isolated from ccRCC cells using the quick RNA extraction kit (Yishan biotechnology). cDNA was synthesized using the All‐in‐one RT SuperMix Perfect for qPCR (Vazyme, Shanghai, Chna). Finally, qRT‐PCR was performed using the ChamQ Universal SYBR qPCR master Mix (Vazyme), primers and diluted cDNA in a Bio‐Rad CFX96 real‐time system according to the manufacturer's protocols. The mRNA expression levels of the genes of interest were first normalized to GAPDH expression, and then the ΔΔCq method was applied to calculate the relative mRNA expression levels of each gene. The primers used in this study were as follows:

RCC1 forward: GGCTTGGTGCTGACACTAGGC.

RCC1 reverse: CCTCCACTGATGTGTCCCTTC.

GAPDH forward: GCACCGTCAAGGCTGAGAAC.

GAPDH reverse: TGGTGAAGACGCCAGTGGA.

EZH2 forward: AGGACGGCTCCTCTAACCAT.

EZH2 reverse: CTTGGTGTTGCACTGTGCTT.

### 2.8 Co‐immunoprecipitation (Co‐IP) assay and ubiquitylation assay

For the Co‐IP assay, 40 μL of protein A magnetic beads (MedChemExpress) was separately incubated with anti‐immunoglobulin G (IgG), anti‐EZH2 (CST), and anti‐RCC1 on a horizontal rotation instrument at 4°C for 12 h. The next day, the beads were successively washed with PBST three times for 5 min each and incubated with lysates from pretreated 786‐O and A498 cells, overnight at 4°C. On the third day, the beads were also washed with PBST three times for 5 min each and mixed with 1X loading buffer (Fdbio) at 100°C for 10 min. Finally, the beads were collected using magnetic separator, and the loading buffer was used for western blotting.

As for the ubiquitylation assay, the cells were treated with 20 μM MG132 (MCE) for 6 h before the CO‐IP assay. Then, 40 μL of protein A magnetic beads was separately incubated with anti‐immunoglobulin G (IgG) and anti‐EZH2 (CST) on a horizontal rotation instrument at 4°C for 12 h. The next day, the beads were successively washed with PBST three times for 5 min each and incubated with pretreated ccRCC cell lysates overnight at 4°C. On the third day, the beads were also washed with PBST three times for 5 min each and mixed with 1X loading buffer (Fdbio) at 100°C for 10 min. The buffer was collected for western blotting. Western blotting was performed using anti‐EZH2 (1:1000, CST) and anti‐UB (1:1000, Abclonal) as the primary antibodies.

### 2.9 Immunofluorescence

A498 and 786‐O cells were fixed using 4% paraformaldehyde for 15 min, washed with PBS three times and then treated simultaneously with 0.5% Triton X 100 and 4% BSA at room temperature for 1 h to improve their membrane permeability and block nonspecific antibody binding. The cells were washed with PBS three times and further incubated with anti‐EZH2 (rabbit antibody, CST) and anti‐RCC1 (murine antibody, Santa Cruz) at 4°C overnight. The next day, the cells were washed again with PBS for three times and subsequently incubated with fluorescent secondary antibodies (Goat Anti‐Rabbit Alexa Fluor 488, Goat Anti‐Mouse Alexa Fluor 594; Fdbio) for 1 hour in combination with DAPI (5 min). After washing three times with PBS, the cells were finally observed using a confocal microscope (Nikon) and photographed.

### 2.10 Protein stability assay

For this assay, 786‐O and A498 cells (pretreated with si‐NC/si‐RCC1 for 48 h) were incubated with 25 μg/mL of cycloheximide (CHX, MCE) for the chosen times. Then, the cells were collected and lysed in RIPA solution (Fdbio) for further western blotting analysis.

### 2.11 Animal assay

BALB/C nude mice were purchased from Gem Pharmatech biotechnology company. Four‐week‐old mice were reared in the pathogen‐free facilities of the First Affiliated Hospital of Zhejiang University. All experimental procedures on mice were approved by the Zhejiang Medical Experimental Animal Care Commission (ST2023006). Before the animal experiments, 786‐O cells (stably transfected with the sh‐NC/sh‐RCC1 lentivirus) were suspended with PBS and adjusted to the concentration of 4 × 10^6^ cells/200 μL. Then, 200 μL of cell suspension was subcutaneously injected into the right flank of each mouse using a 2 mL syringe. Every 10 days, the tumor volumes were measured. All nude mice were sacrificed by cervical dislocation after 40 days. The length, width, and weight of the tumors were recorded, and the tumor volumes were calculated using a formula (0.5 × length × width^2^).

### 2.12 Statistics

Graphpad Prism8 software (Graphpad Software) and SPSS 22.0 (IBM Corp.) were chosen to analyze the differences between different groups. The data were collected from three independent experiments and are presented as the mean ± standard deviation. The Kolmogorov–Smirnov test was initially used to test the normality of the data. A Pearson's chi‐squared test or a continuity correction chi‐squared test was conducted to analyze the relationships between RCC1 expression and clinicopathological features. As for the CCK8 assay, two‐way ANOVA was used. To analyze the differences in cell cycle distribution, apoptosis, colony formation, tumor weight, and volume, the unpaired‐sample two‐sided *t*‐test was chosen. The Mann–Whitney *U*‐test was used to analyze the IHC results; *p* < 0.05 was considered as statistically significant.

## 3 RESULTS

### 3.1 RCC1 expression is highly elevated in ccRCC samples and correlates with worse prognosis and clinicopathological features

**FIGURE 1:**
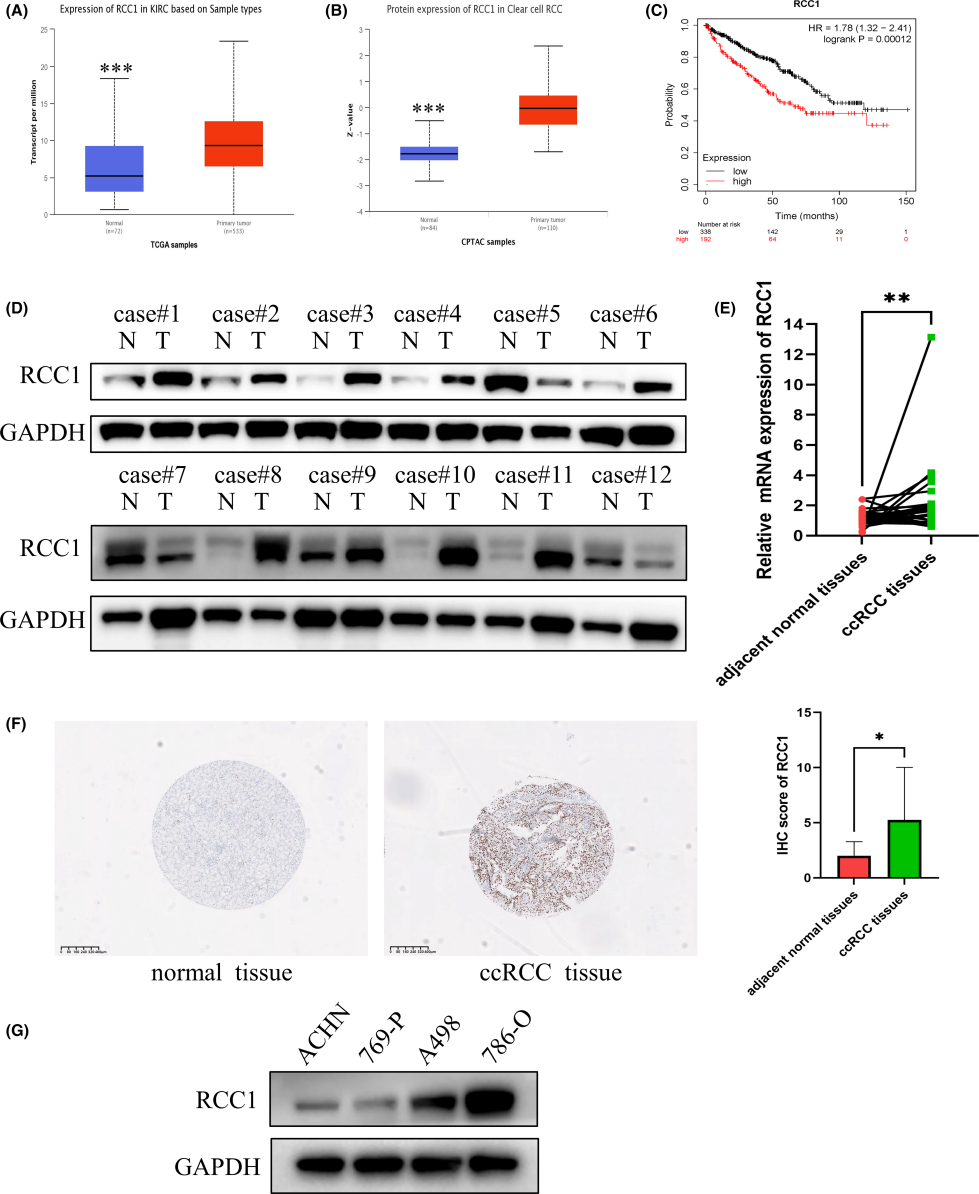
RCC1 is overexpressed in ccRCC and associated with worse patient survival. (A‐B) The expression of RCC1 (A) mRNA and (B) protein was explored using the UALCAN database. (C) A high expression of RCC1 was associated with worse overall survival (KM plotter). (D) The protein expression level of RCC1 was verified in 12 paired clinical ccRCC specimens. (E) The mRNA expression level of RCC1 was detected in 30 paired clinical ccRCC specimens. (F) The expression level of RCC1 was detected in other 30 paired ccRCC samples using immunohistochemical staining. Representative pictures of RCC1 staining of normal and tumor samples. (G) The protein expression of RCC1 in various ccRCC cell lines. *p < 0.05, **p < 0.01, ***p < 0.001.

The UALCAN database was used to confirm that in the TCGA dataset, RCC1 was overexpressed at both the mRNA and protein levels in ccRCC samples compared to normal tissues (Figure 1A,B). In addition, using the KM plotter portal, we found that patients possessing a relatively high RCC1 mRNA level had worse overall survival according to the TCGA dataset (Figure 1C). Consistent with the dataset results, the mRNA and protein expression levels of RCC1 were mostly higher in ccRCC samples than in adjacent normal tissues, as confirmed by WB, IHC, and qRT‐PCR assays (Figure 1D–F). We also explored the relationship between RCC1 expression and clinical characteristics and found that RCC1 expression was positively correlated with nodal metastasis, tumor grade, and TNM stage in the TCGA dataset (Figure S1A–D and Table 1). Finally, we analyzed the expression of RCC1 in various ccRCC cells to choose the proper cell lines for knockdown and overexpression assays (Figure 1G). The cell lines 786‐O and A498 were chosen to perform knockdown assays due to their relatively high protein expression of RCC, while 769‐P cells were chosen to perform overexpression assays due to their relatively low protein expression of RCC1. The above‐reported results demonstrated that a high RCC1 expression might indicate a poor prognosis for ccRCC patients.

**TABLE 1:** The relationship between RCC1 mRNA expression and different clinicopathological characteristics in TCGA database.

Characteristics	Low expression of RCC1	High expression of RCC1	*p* value
*n*	266	266	
Pathologic T classification, *n* (%)			<0.001
T2 & T1	193 (36.3%)	148 (27.8%)	
T4 & T3	73 (13.7%)	118 (22.2%)	
Pathologic N classification, *n* (%)			0.004
N0	134 (52.3%)	106 (41.4%)	
N1	3 (1.2%)	13 (5.1%)	
Pathologic M classification, *n* (%)			<0.001
M0	223 (44.6%)	198 (39.6%)	
M1	25 (5%)	54 (10.8%)	
Histologic grade, *n* (%)			<0.001
G1 & G2	140 (26.7%)	102 (19.5%)	
G3 & G4	119 (22.7%)	163 (31.1%)	
Age, *n* (%)			0.603
≤60	129 (24.2%)	135 (25.4%)	
>60	137 (25.8%)	131 (24.6%)	

### 3.2 Knockdown of RCC1 inhibits ccRCC cell growth, and overexpression of RCC1 promotes ccRCC cell proliferation

**FIGURE 2:**
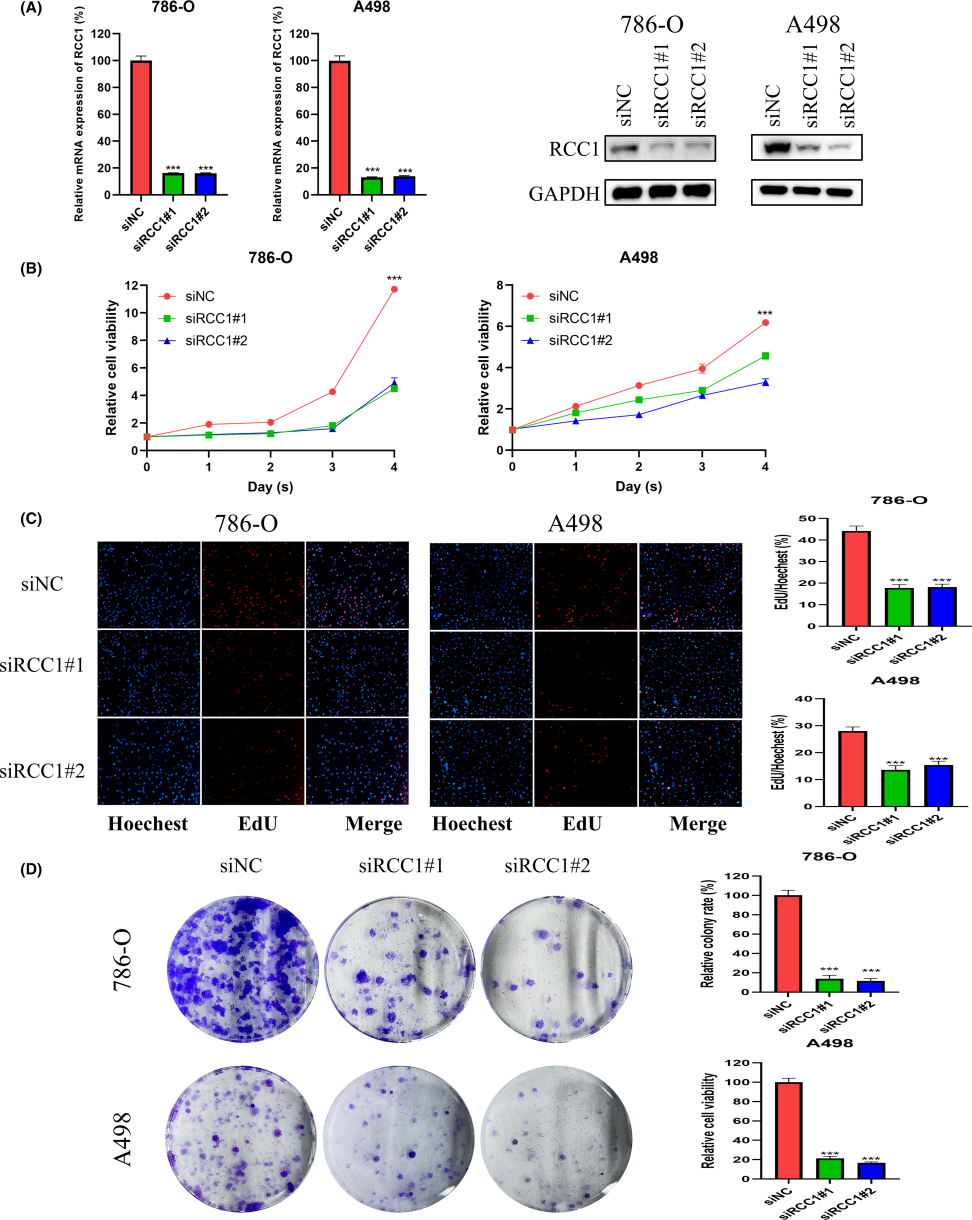
RCC1 knockdown impaired the proliferation of ccRCC cells in vitro. (A) The efficiency of RCC1 knockdown was confirmed at both mRNA and protein levels. (B) The effect of RCC1 knockdown on the proliferation of 786‐O and A498 cells was detected by the CCK8 assay. (C) The effect of RCC1 knockdown on the proliferation of 786‐O and A498 cells was detected by the EDU assay. Magnification: 100×. (D) The effect of RCC1 knockdown on the proliferation of 786‐O and A498 cells was detected by the colony formation assay. *p < 0.05, **p < 0.01, ***p < 0.001.

We first verified the knockdown efficiency of RCC1 in 786‐O and A498 cells and found that si‐RCC1#1 and si‐RCC1#2 significantly reduced the mRNA and protein expression levels of RCC1 (Figure 2A). The CCK‐8 assay showed that the knockdown of RCC1 obviously reduced ccRCC cell proliferation (Figure 2B), while the overexpression of RCC1 in 769‐P cells accelerated cell growth (Figure S2B). Moreover, the colony formation assay and the EDU assay also demonstrated that the knockdown of RCC1 in two cell lines hindered cell growth and viability (Figure 2C,D), while RCC1 overexpression promoted cell cycle progression (Figure S2B).

### 3.3 RCC1 is essential for ccRCC cell cycle and inhibits cell apoptosis

**FIGURE 3:**
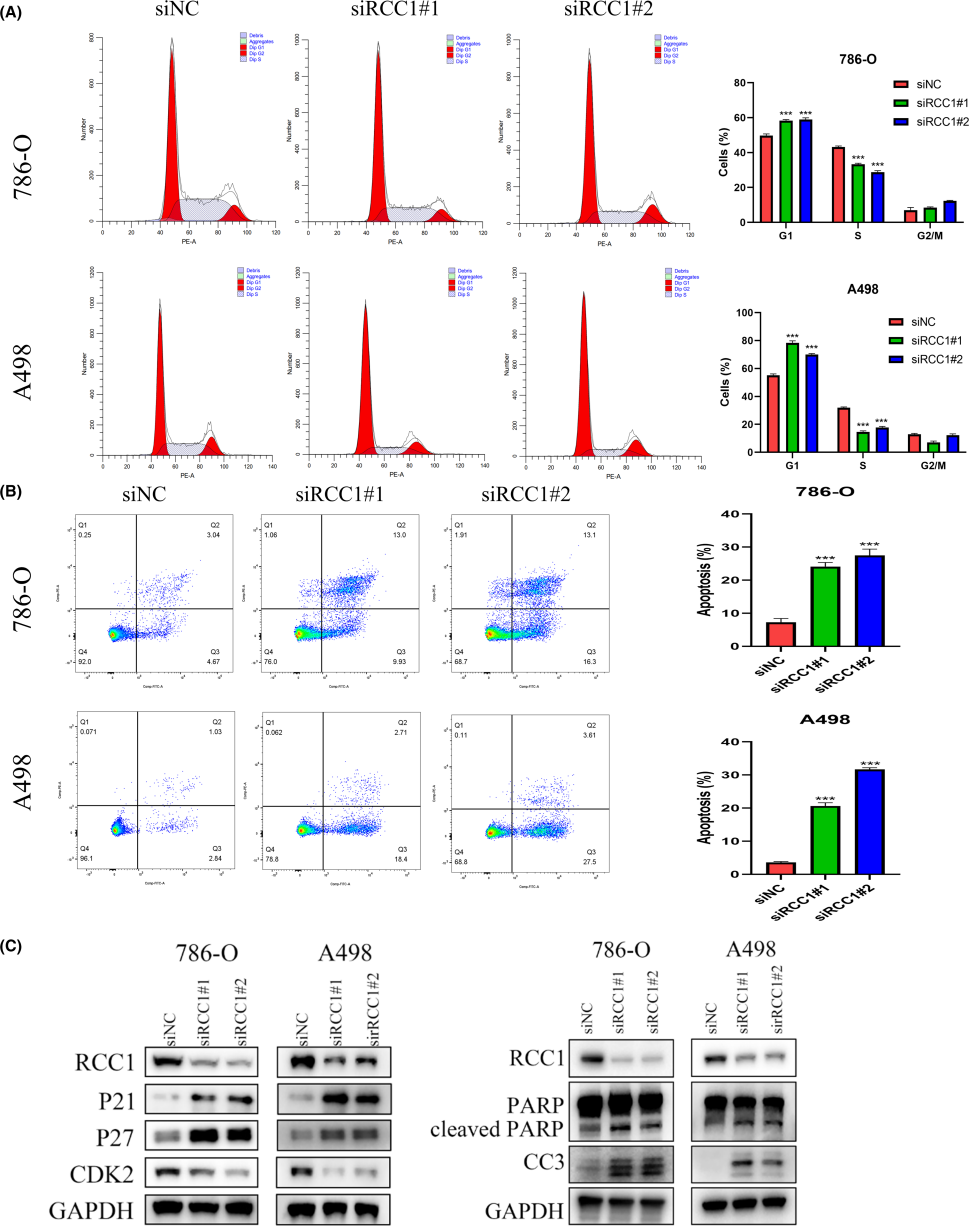
RCC1 knockdown induced cell cycle arrest and apoptosis in ccRCC cells in vitro. (A) The effect of RCC1 knockdown on the apoptosis of 786‐O and A498 cells was detected by FACS. (B) The effect of RCC1 knockdown on the cell cycle distribution of 786‐O and A498 cells was detected by FACS. (C) The effect of RCC1 knockdown on cell cycle‐ and apoptosis‐related genes was detected by western blotting. GAPDH was used as a control. *p < 0.05, **p < 0.01, ***p < 0.001.

Compared with control cells (transfected with si‐NC), 786‐O and A498 cells transfected with si‐RCC1 showed changes in cell cycle distribution, with more cells arrested in the G0/G1 phase and fewer cells transitioned to the S phase (Figure 3A). The overexpression of RCC1 in 769‐P cells accelerated the G1 phase transition (Figure S2C). We also explored the effect of RCC1 on cell apoptosis. The results revealed that the knockdown of RCC1 induced a significant increase in apoptosis in 786‐O and A498 cells (Figure 2B), while the overexpression of RCC1 suppressed apoptosis (Figure S2D). In addition, the expression of cell cycle‐ and apoptosis‐related genes was detected after knockdown or overexpression of RCC1 using WB (Figure 2C, Figure S2E). The expression of P21, P27, cleaved PARP, and cleaved caspase 3 protein was increased, and that of CDK2 protein was decreased in cells transfected with si‐RCC1, while the overexpression of RCC1 led to opposite results.

### 3.4 RCC1 regulates EZH2 expression at the posttranscriptional level

**FIGURE 4:**
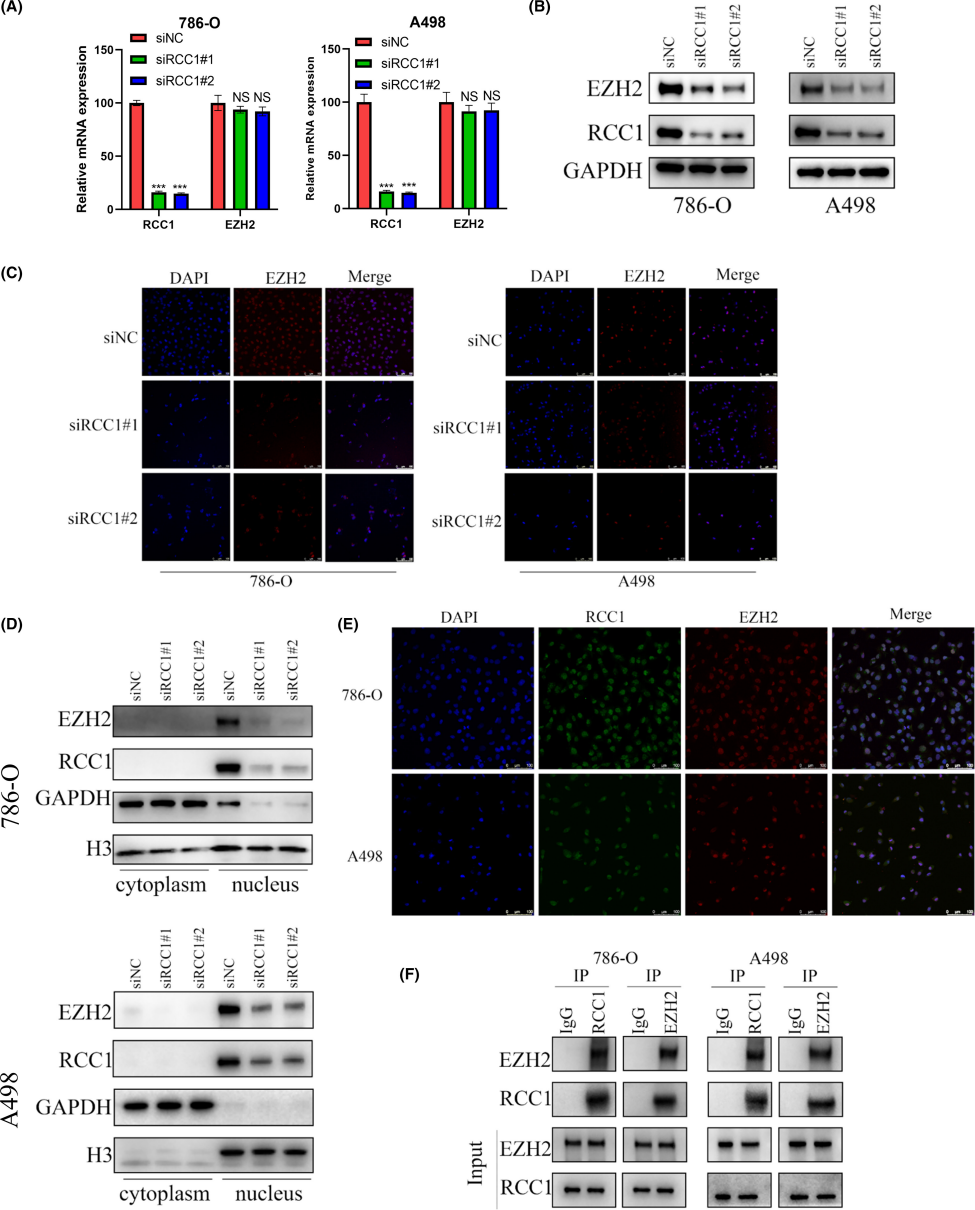
RCC1 regulated EZH2 expression at the post‐transcriptional level. (A) RCC1 knockdown did not influence the mRNA expression of EZH2. (B) RCC1 knockdown influenced the protein expression of EZH2. (C) The effect of RCC1 knockdown on EZH2 protein expression was detected by IF. (D) The effect of RCC1 knockdown on the location and expression of EZH2 was detected by nuclear and cytoplasmic separation experiments. GAPDH was used as a cytoplasmic control, and H3 as a nuclear control. (E) The localization of RCC1 and EZH2 was detected by IF. DAPI was used as a positive control for nuclear localization. Scale bar:100 μm. (F) The interaction of the RCC1 and EZH2 proteins was detected by Co‐Ip. ***p < 0.001.

We used the BioGRID portal^37^ (https://thebiogrid.org/, accessed on August 12, 2021) to identify proteins that could interact with RCC1. The BioGRID portal is a platform that integrates published mass spectrometry data and helps find interacting proteins, requiring only the protein name. Interestingly, the binding of EZH2, a well‐known tumor‐promoting protein, to was found to be highly likely. Then, we detected EZH2 mRNA and protein expression levels after knockdown or overexpression of RCC1 using qRT‐PCR and WB (Figure 4A,B, Figure S2F,G). The results showed that RCC1 did not affect the mRNA level of EZH2 but regulated its protein level. IF analysis further confirmed that the fluorescence intensity associated with the EZH2 protein was decreased after RCC1 knockdown (Figure 4C). Moreover, we also found that the nuclear levels of the EZH2 protein were significantly reduced after RCC1 knockdown, as shown by analysis with the Nuclei and Cytoplasmic Protein Extraction kit (Figure 4D). The similar nuclear location of RCC1 and EHZ2 in 786‐O and A498 cell lines provided evidence for their potential interaction (Figure 4E). In addition, the Co‐IP assay further proved that RCC1 could bind to EZH2 (Figure 4F). The correlation between RCC1 and EZH2 expression in ccRCC samples was also explored using IHC (Figure S3A,B). The results showed that the protein expression of RCC1was highly correlated (*R* = 0.6061, *p* = 0.0004) to that of EZH2.

### 3.5 RCC1 knockdown reduces EZH2 protein stability

**FIGURE 5:**
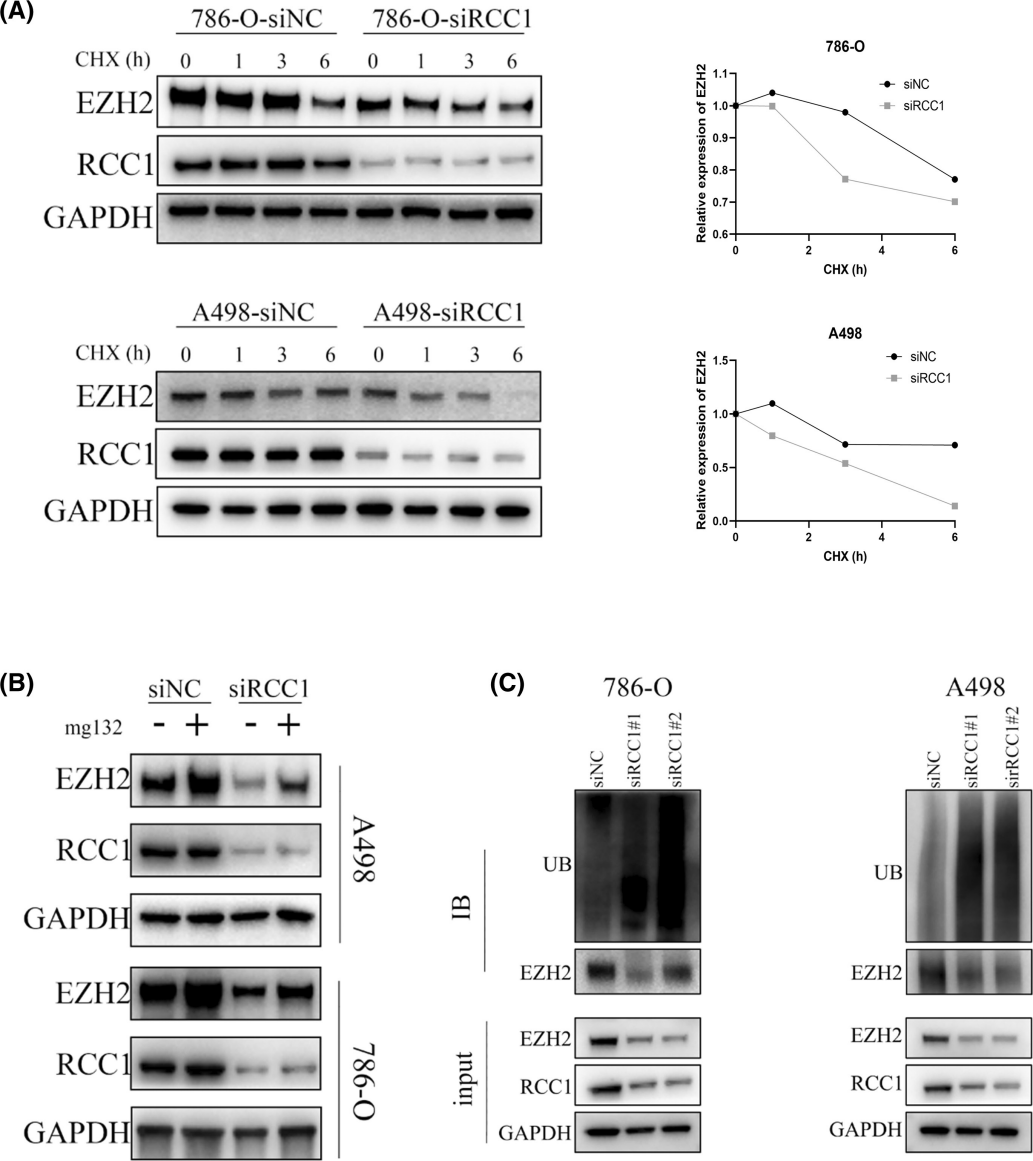
RCC1 regulated EZH2 expression partially through the ubiquitin–proteasome pathway. (A) RCC1 knockdown decreased the protein stability of EZH2. We used 20 μg/mL of CHX in the protein degradation assay. (B) The EZH2 protein degradation caused by RCC1 knockdown was partially reversed by inhibiting the proteasome pathway. To inhibit the proteasome pathway, the cells were treated with 20 μM MG132 for 6 h. (C) RCC1 knockdown promoted EZH2 protein ubiquitylation.

Our previous results showed that RCC1 regulated EZH2 expression at the posttranscriptional level; so, we next explored how RCC1 regulated EZH2 protein expression. The CHX protein stability assay revealed that the knockdown of RCC1 significantly promoted the time‐dependent degradation of EZH2 protein compared with control cells (Figure 5A). The proteasome pathway is one of most important signaling cascades that control protein degradation depending on ATP availability. We used MG132 to inhibit the proteasome pathway and found that the reduction of EZH2 protein caused by RCC1 knockdown was partly reversed (Figure 5B). A ubiquitylation assay revealed that RCC1 knockdown facilitated EZH2 degradation by boosting EZH2 ubiquitination (Figure 5C).

### 3.6 Overexpression of EZH2 partly rescues the tumor suppression effect caused by RCC1 knockdown in vitro

**FIGURE 6:**
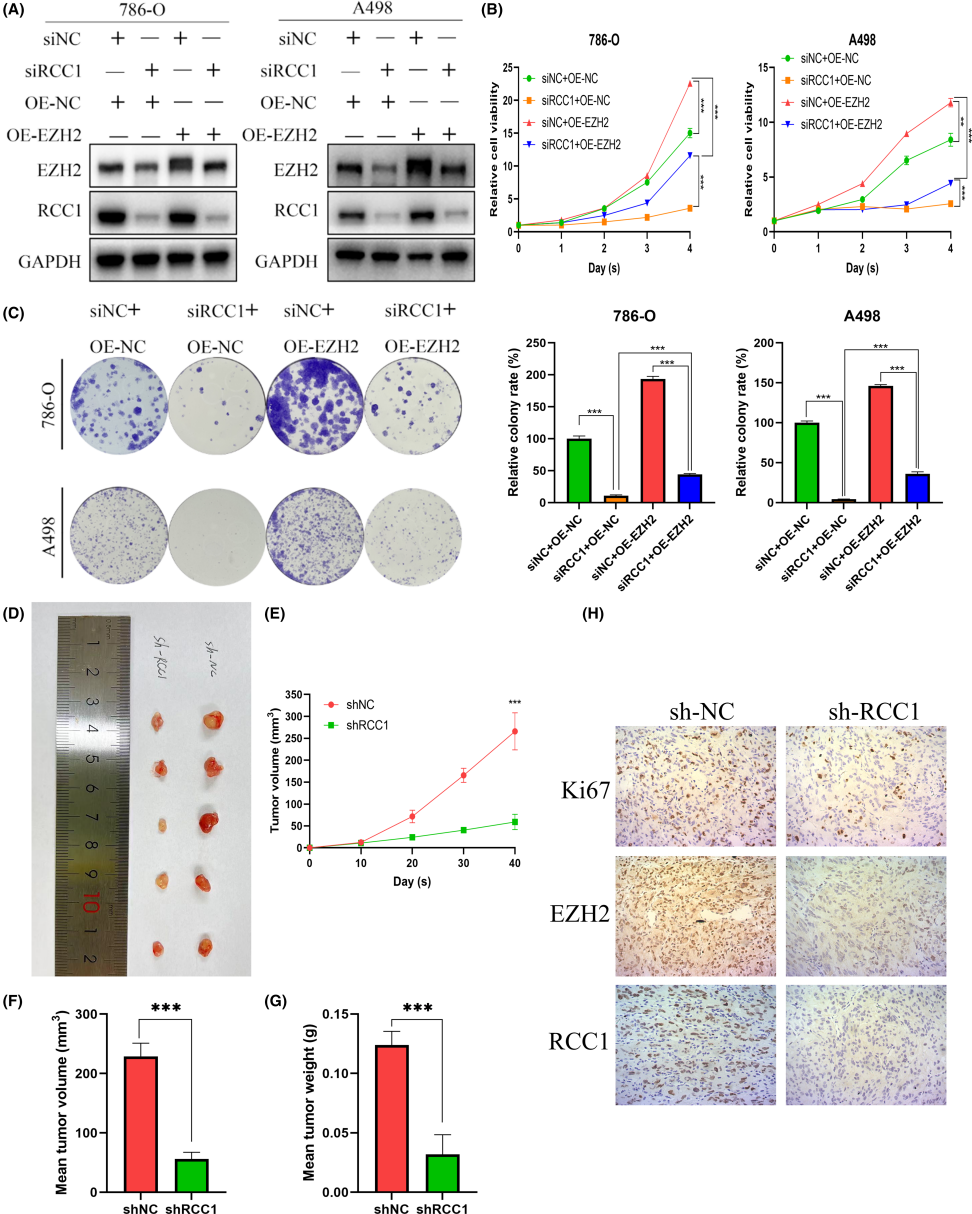
Overexpression of EZH2 partly rescued the tumor suppression effect caused by RCC1 knockdown, and RCC1 knockdown inhibited tumorigenesis in vivo. (A) The protein levels of EZH2 and RCC1 were measured by western blot in 786‐O and A498 cells transfected with siRCC1 and/or a plasmid carrying EZH2. (B) Overexpression of EZH2 partly rescued the tumor suppression effect caused by RCC1 knockdown, as confirmed by the CCK8 assay. (C) Overexpression of EZH2 partly rescued the tumor suppression effect caused by RCC1 knockdown, as confirmed by the colony formation assay. (D) General view of the tumors in the sh‐NC and sh‐RCC1 groups. (E) The sh‐RCC1 tumors grew more slowly in vivo compared with the sh‐NC tumors. (F) The sh‐RCC1 tumors were smaller compared with the sh‐NC tumors. (G) The sh‐RCC1 tumors were lighter compared with the sh‐NC tumors. (H) Representative IHC pictures of Ki67, EZH2 and RCC1 in sh‐NC and sh‐RCC1 tumors. Magnification: 100×. ***p < 0.001.

The WB assay confirmed that the overexpression of EZH2 successfully reversed the EZH2 reduction caused by RCC1 knockdown (Figure 6A). The CCK8 assay and colony formation assay were then performed to verify whether RCC1 regulated ccRCC cell growth through EZH2 (Figure 6B,C). The results indicated that EZH2 overexpression significantly inhibited the negative effects on cell growth and colony number caused by RCC1 knockdown.

### 3.7 RCC1 knockdown suppresses tumor progression in vivo

We injected 786‐O cells pretreated with sh‐NC or sh‐RCC1 lentivirus in the right flank of mice and let them grow for 40 days. The tumors were extracted and photographed on Day 40 (Figure 6D). Compared with tumors treated with sh‐NC, the tumors treated with sh‐RCC1 grew more slowly and reached a lower tumor weight and a smaller tumor volume (Figure 6E–G). The subsequent IHC assay demonstrated that the sh‐RCC1 groups had lower EZH2 and ki‐67 expression compared with the corresponding control groups (Figure 6H). These results indicated that RCC1 knockdown may regulate EZH2 expression to suppress tumor growth in vivo.

## 4 DISCUSSION

ccRCC is characterized be intrinsic resistance to chemotherapy and radiotherapy^4^, ^7^; therefore, great efforts are being made to develop targeted therapies. Although the existent antiangiogenic treatments (sorafenib and axitinib) and immunotherapies (tislelizumab, pembrolizumab, and nivolumab) have prolonged and improved the survival and prognosis of metastatic ccRCC patient,^9^, ^10^, ^11^ many patients eventually progress, becoming resistant to targeted therapies.^38^, ^39^, ^40^ Therefore, it is urgent to discover novel drug targets in ccRCC.

RCC1 facilitates the transformation of RanGDP to RanGTP, thereby affecting a series of normal cell physiological activities like nuclear transport and spindle and nuclear envelope formation.^15^, ^19^ Recently, multiple studies reported that aberrant expression of RCC1 was involved in tumorigenesis.^17^, ^18^, ^19^, ^20^, ^21^ However, the expression profile and underlying function of RCC1 in ccRCC are not known. In the current study, we found that RCC1 was mostly overexpressed in ccRCC tissues compared with adjacent normal tissues, which indicated that RCC1 may act as a tumor promoter. TCGA data visualized by the GEPIA and UALCAN portals showed that patients with high TNM stage and poor tumor grade had high expression levels of RCC1. Moreover, the patients with a relatively high expression level of RCC1 had shorter overall survival.

Further functional experiments confirmed that RCC1 knockdown suppressed tumor growth and induced cell apoptosis and G1 phase arrest, while the overexpression of RCC1 had opposite effects and facilitated tumor progression. P21and P27 acting as CDK inhibitors play a significant role in controlling the G1/S transition.^41^ Therefore, we hypothesized that the G1 phase arrest caused by RCC1 knockdown was most likely regulated by P21 and P27. Western blotting analyses showed that the expression of the G1 phase‐arrest‐related proteins P21 and P27 increased and that of CDK2 decreased when repressing RCC1, whereas the overexpression of RCC1 led to opposite results. As for mitochondrial apoptosis, caspase‐3 acts as downstream apoptosis mediator and promotes PARP degradation.^42^ The amounts of cleaved caspase‐3 and PARP reflect the extent of apoptosis. In our study, RCC1 knockdown upregulated the levels of cleaved caspase‐3 and cleaved PARP, while RCC1 overexpression led to their downregulation. These results indicated that RCC1 exerted a tumor promotion effect by regulating mitochondrial‐mediated apoptosis.

EZH2 was shown to participate in the development and metastasis formation of various tumors.^24^, ^25^, ^26^, ^27^, ^28^, ^29^, ^33^ In ccRCC, EZH2 could be an independent prognostic marker.^43^ Silencing EZH2 expression weakened the proliferation capacity of ccRCC cells through the induction of G1 phase arrest and apoptosis.^28^ Moreover, it was reported that EZH2 could catalyze the formation of H3K37me3 in the promoter of P21 and P27 to repress the expression of these genes.^44^, ^45^, ^46^ In this study, we found that RCC1 regulated EZH2, influencing the progression of ccRCC. In detail, the expression of genes downstream of EZH2 like P21 and P27 was increased or decreased after RCC1 knockdown or overexpression. In addition, the tumor suppression effect caused by RCC1 knockdown could be rescued by EZH2 overexpression in vitro. Further investigation showed that RCC1 could directly bind EZH2 and improve its protein stability rather than increase its expression by acting at the transcription level. The C‐terminus of RCC1 contains the RCC1‐like domain (RLD) which contains the seven‐blade β propeller domain.^47^ Each blade in the β propeller structure consists of 51–68 residue repeats and forms four antiparallel chains with loops between them. The β propeller structure is involved in protein–protein interactions^47^, ^48^, ^49^ and thus provides a possibility of interaction between RCC1 and EZH2. The ubiquitin–proteasome pathway was extensively reported to be involved in the pathogenesis of certain malignancies by mediating the degradation of proteins playing a role in these pathologies or reducing their stability.^50^, ^51^, ^52^ In this pathway, substrates, linked to various 76‐amino acid ubiquitin monomers, are recognized and processed by the 26S proteasome. Recently, it was found that ubiquitination occurs not only in the cytoplasm but also in the nucleus. For example, the ubiquitination of EZH2 could be regulated by the E3 ubiquitin ligases SMURF2 and FBXW7 in the nucleus.^53^, ^54^, ^55^ In the current study, we discovered that after RCC1 knockdown, EZH2 was linked to more ubiquitin residues, as confirmed by CO‐IP assays. In addition, the reduction of the EZH2 protein caused by RCC1 silencing could be reversed after adding MG132 (an inhibitor of the proteasome pathway). To summarize, RCC1 improved EZH2 protein stability through the ubiquitin–proteasome pathway. Whether RCC1 affected EZH2 ubiquitination by competing with E3 ubiquitin ligases needs further exploration.

In conclusion, we first identified the expression profile and clinical value of RCC1 in ccRCC. Further functional verification and mechanism investigation uncovered that RCC1 affected the progression of ccRCC by regulating EZH2 protein stability. Moreover, in vivo animal experiments proved that targeting RCC1 could significantly restrain tumor growth, providing evidence for the potential therapeutic value of RCC1 in ccRCC.

## AUTHOR CONTRIBUTIONS

**Yunfei Wu:** Conceptualization (equal); writing – original draft (equal). **Zhijie Xu:** Conceptualization (equal); methodology (equal); visualization (equal). **Xiaoyi Chen:** Formal analysis (equal); investigation (equal). **Guanghou Fu:** Methodology (equal); validation (equal). **Junjie Tian:** Methodology (equal); validation (equal). **Baiye Jin:** Funding acquisition (equal); writing – review and editing (equal).

## CONFLICT OF INTEREST STATEMENT

The authors declare no conflicts of interest.

## ETHICAL APPROVAL STATEMENT

Adjacent normal tissues and renal cell carcinoma samples were collected from the archive of the Institute of Urology, the First Affiliated Hospital of Zhejiang University School of Medicine. Our study was carried out in accordance with the Declaration of Helsinki and approved by the Ethics Committee of the First Affiliated Hospital at Zhejiang University School of Medicine (IIT20200733A). All patients provided a written informed consent. The animal experiments were approved by the Zhejiang University experimental animal welfare ethics review committee (ST2023006). The animal experiments were also conducted under our institutional guidelines and regulations for caring and using animals.

## Supporting information


Figure S1: RCC1 expression is positively correlated with poor clinicopathologic features. (A) The mRNA expression levels of RCC1 were positively related with the nodal metastasis status. (B) The mRNA expression levels of RCC1 were positively related with the histologic grade. (C) The protein expression levels of RCC1 were positively related with the histologic grade. (D) The protein expression levels of RCC1 were positively related with the pathological stage. *** p < 0.001.

Figure S2: RCC1 overexpression promoted tumorigenesis of ccRCC cells in vitro. (A) The effect of RCC1 overexpression on the proliferation of 769‐P cells was detected by the CCK8 assay. (B) The effect of RCC1 overexpression on the proliferation of 769‐P cells was detected by the colony formation assay. (C) The effect of RCC1 overexpression on the cell cycle distribution of 769‐P cells was detected by FACS. (D) The effect of RCC1 overexpression on the apoptosis of 769‐P cells was detected by FACS. (E) The effect of RCC1 overexpression on cell cycle‐ and apoptosis‐related genes was detected by Western blotting. (F‐G) The effect of RCC1 overexpression on EZH2 was detected at the mRNA and protein expression levels. ** p < 0.01, *** p < 0.001.

Figure S3: The correlation between RCC1 and EZH2 expression in ccRCC tissues. (A) IHC was performed to verify the correlation between RCC1 and EZH2 expression in ccRCC tissues (n = 30). Representative pictures of RCC1 and EZH2 staining. (B) The statistical data showed that the expression of EZH2 was closely associated with RCC1 expression (R = 0.6061, p = 0.0004).

## Data Availability

KM plotter (http://kmplot.com/analysis/), UALCAN (http://ualcan.path.uab.edu/index.html/) and The Cancer Genome Atlas (TCGA) database (https://cancergenome.nih.gov/) are open source online datasets. Informed consent is not required for publicly available datasets.
