# Supplementary figures and images for: RCC1 functions as a tumor facilitator in clear cell renal cell carcinoma by dysregulating cell cycle, apoptosis, and EZH2 stability
Source: Cancer Med. 2023 Sep 25;12(19):19889–903. doi: 10.1002/cam4.6594 (PMC10587970; doi:10.1002/cam4.6594)

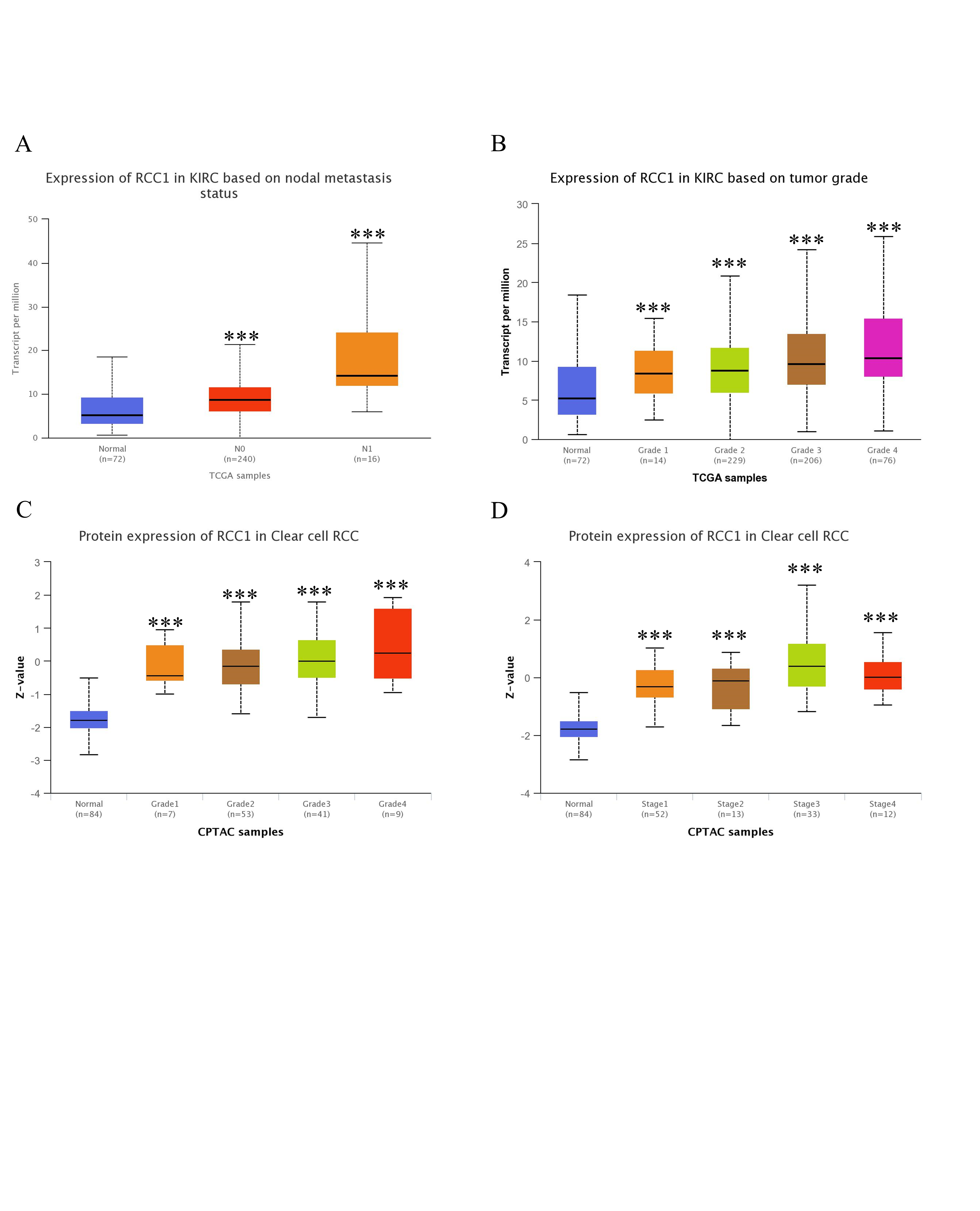

Supplement: Supplementary file 1 — Figure S1: RCC1 expression is positively correlated with poor clinicopathologic features. (A) The mRNA expression levels of RCC1 were positively related with the nodal metastasis status. (B) The mRNA expression levels of RCC1 were positively related with the histologic grade. (C) The protein expression levels of RCC1 were positively related with the histologic grade. (D) The protein expression levels of RCC1 were positively related with the pathological stage. *** p < 0.001. [file CAM4-12-19889-s002.tif]

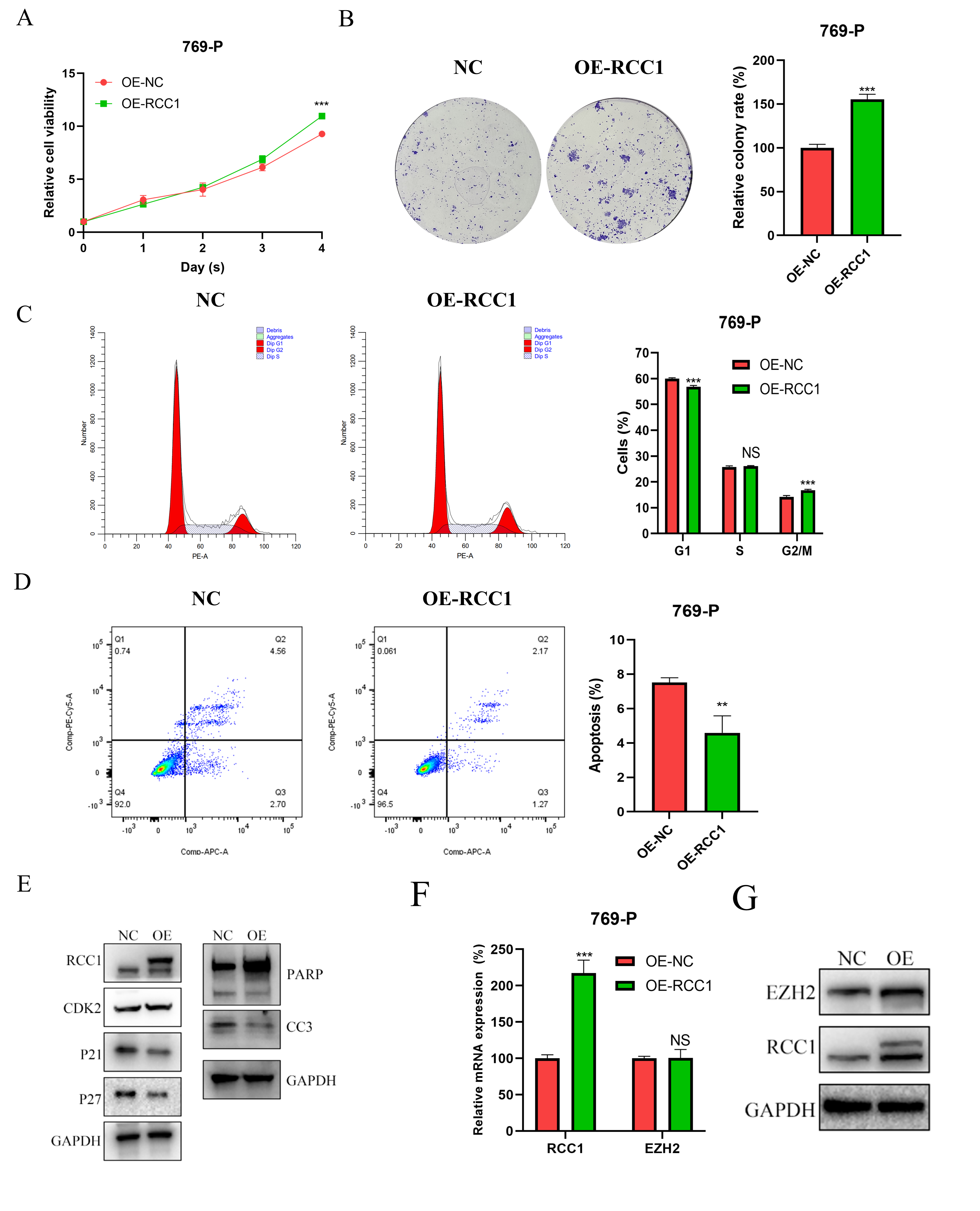

Supplement: Supplementary file 2 — Figure S2: RCC1 overexpression promoted tumorigenesis of ccRCC cells in vitro. (A) The effect of RCC1 overexpression on the proliferation of 769‐P cells was detected by the CCK8 assay. (B) The effect of RCC1 overexpression on the proliferation of 769‐P cells was detected by the colony formation assay. (C) The effect of RCC1 overexpression on the cell cycle distribution of 769‐P cells was detected by FACS. (D) The effect of RCC1 overexpression on the apoptosis of 769‐P cells was detected by FACS. (E) The effect of RCC1 overexpression on cell cycle‐ and apoptosis‐related genes was detected by Western blotting. (F‐G) The effect of RCC1 overexpression on EZH2 was detected at the mRNA and protein expression levels. ** p < 0.01, *** p < 0.001. [file CAM4-12-19889-s001.tif]

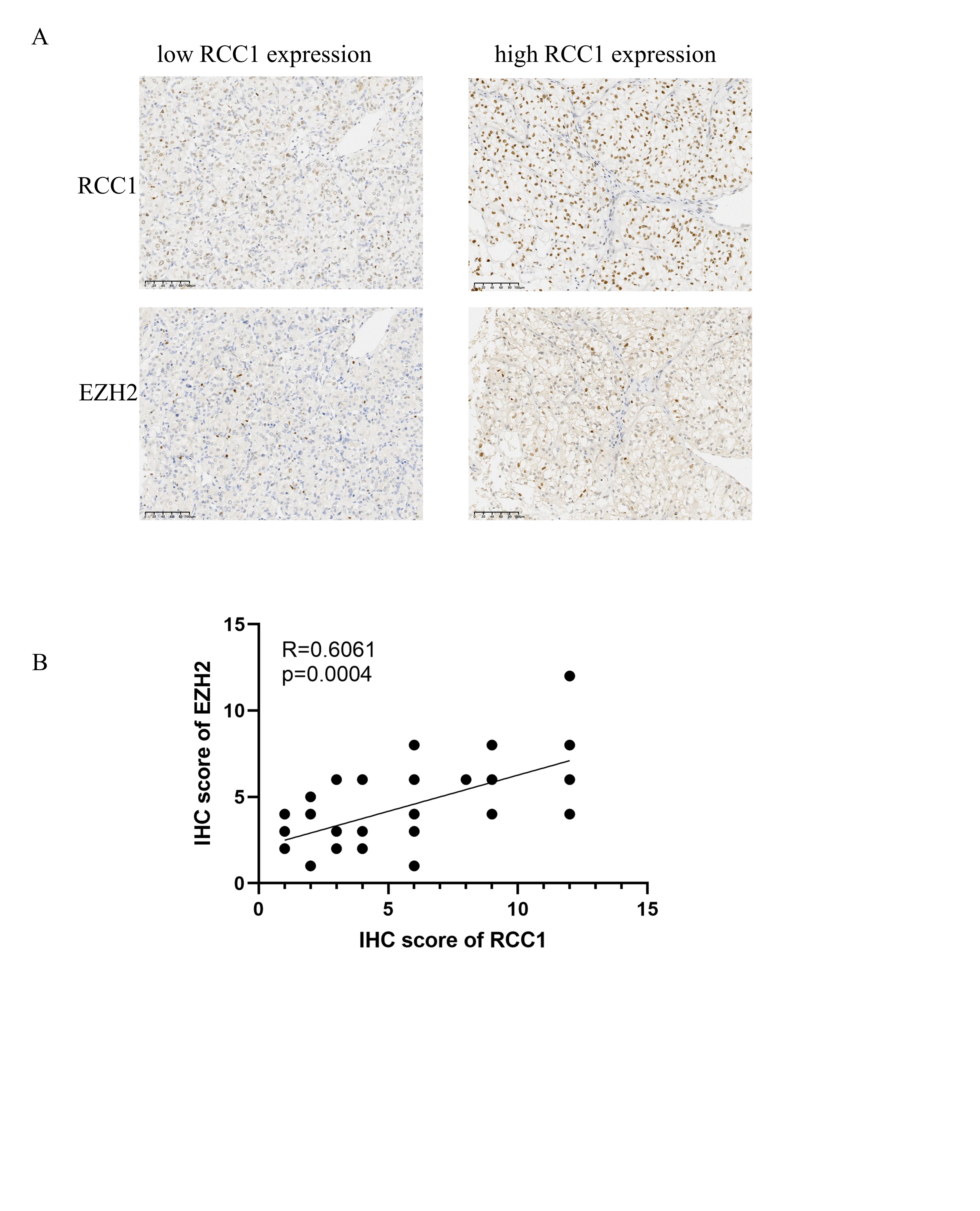

Supplement: Supplementary file 3 — Figure S3: The correlation between RCC1 and EZH2 expression in ccRCC tissues. (A) IHC was performed to verify the correlation between RCC1 and EZH2 expression in ccRCC tissues (n = 30). Representative pictures of RCC1 and EZH2 staining. (B) The statistical data showed that the expression of EZH2 was closely associated with RCC1 expression (R = 0.6061, p = 0.0004). [file CAM4-12-19889-s003.tif]
